# Rapid Intervention to Reduce Ebola Transmission in a Remote Village — Gbarpolu County, Liberia, 2014

**Published:** 2015-02-27

**Authors:** David J. Blackley, Kim A. Lindblade, Francis Kateh, Laura N. Broyles, Matthew Westercamp, John C. Neatherlin, Satish K. Pillai, Anthony Tucker, Joshua A. Mott, Henry Walke, Tolbert Nyenswah

**Affiliations:** 1 Epidemic Intelligence Service, CDC; 2 Influenza Division, National Center for Immunization and Respiratory Diseases, CDC; 3 Liberia Ministry of Health and Social Welfare; 4 Division of Global HIV/AIDS, Center for Global Health, CDC; 5 Division of Global Health Protection, Center for Global Health, CDC; 6 Division for Preparedness and Emerging Infections, National Center for Emerging and Zoonotic Infectious Diseases, CDC; 7 Division of High-Consequence Pathogens and Pathology, National Center for Emerging and Zoonotic Infectious Diseases, CDC

As late as September 14, 2014, Liberia’s Gbarpolu County had reported zero cases of Ebola virus disease (Ebola) (1). On October 25, the Bong County Health Team, a local health department in the Liberian Ministry of Health and Social Welfare (MOHSW), received confirmation of Ebola in a man who had recently left Geleyansiesu, a remote village of approximately 800 residents, after his wife and daughter had died of illnesses consistent with Ebola. MOHSW requested assistance from CDC, the World Health Organization, and other international partners to investigate and confirm the outbreak in Geleyansiesu and begin interventions to interrupt transmission. A total of 22 cases were identified, of which 18 (82%) were laboratory confirmed by real-time polymerase chain reaction. There were 16 deaths (case-fatality rate = 73%). Without road access to or direct telecommunications with the village, interventions had to be tailored to the local context. Public health interventions included 1) education of the community about Ebola, transmission of the virus, signs and symptoms, the importance of isolating ill patients from family members, and the potential benefits of early diagnosis and treatment; 2) establishment of mechanisms to alert health authorities of possibly infected persons leaving the village to facilitate safe transport to the closest Ebola treatment unit (ETU); 3) case investigation, contact tracing, and monitoring of contacts; 4) training in hygienic burial of dead bodies; 5) active case finding and diagnosis; and 6) isolation and limited no-touch treatment in the village of patients unwilling or unable to seek care at an ETU. The findings of this investigation could inform interventions aimed at controlling focal outbreaks in difficult-to-reach communities, which has been identified as an important component of the effort to eliminate Ebola from Liberia (2).

## Investigation Results

**FIGURE 1:**
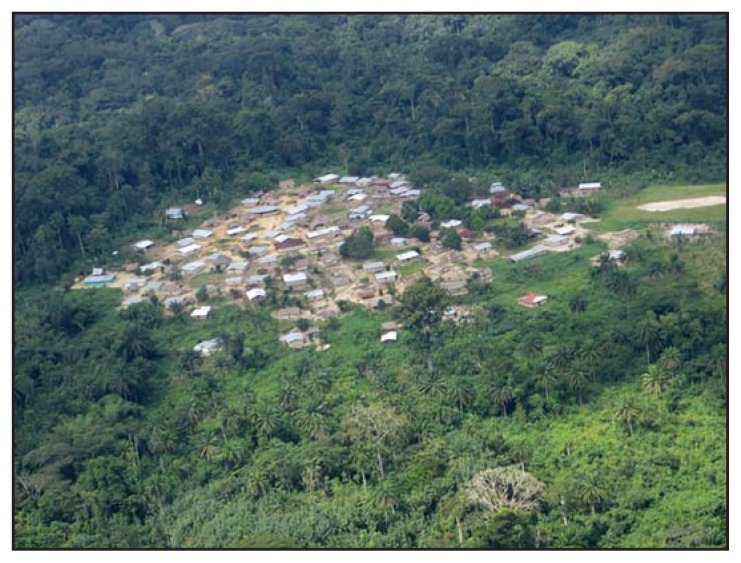
Aerial view of the village of Geleyansiesu — Gbarpolu County, Liberia, November 9, 2014 Photo/Kim A. Lindblade

On September 16, a girl aged 10 years (source patient) attending school in Kakata, Margibi County, returned to Geleyansiesu (southeastern Gbarpolu County, bordering Bong County) (Figure 1), a remote village accessible only by canoe and several hours walking. It was reported by the community that the aunt with whom the source patient resided had recently died of an illness consistent with Ebola. The child became ill on September 18 and was cared for by her stepmother (aged 37 years) before dying on September 27 in Geleyansiesu. At least 13 village residents in addition to the stepmother participated in her burial, none of whom contracted Ebola. The stepmother experienced symptoms consistent with Ebola on October 8, and became the only known patient with infection attributable to the source patient. The severely ill stepmother was carried in a hammock stretcher by at least nine persons from Geleyansiesu to a nearby town to seek medical care; she died on October 11. One of the hammock carriers, her husband (aged 39 years), traveled to a quarantine center for Ebola patient contacts in Gbarnga, Bong County, along with seven family members who had not participated in her transport. Eight of the carriers returned to Geleyansiesu, and none became ill with Ebola. The husband experienced symptoms including fever, vomiting, and diarrhea beginning on October 24 and was transported to the Bong County ETU (Bong ETU) on October 25, where he tested positive for Ebola virus. He recovered and was discharged on November 12; none of the seven immediate family members staying with him in the quarantine center became ill.

On October 30, MOHSW, Gbarpolu County, and Bong County, CDC, and other international partners conducted a brief overnight assessment visit to Geleyansiesu. The purpose of the visit was to determine whether there was ongoing transmission in the village and gather situational information to mount a coordinated public health response, which was complicated by the difficult access to the community. During the visit, team members educated the community about the signs and symptoms of Ebola and the importance of early identification and treatment, along with the options for diagnosis and treatment at the Bong ETU. Although information provided by the community did not suggest any current cases or contacts of the previously identified cases in the village, they did report two recent deaths on October 27 (of farmer A) and October 28 (of farmer B). Farmer A was reported to have died of an injury, whereas farmer B’s death was unexplained. At the time, neither could be linked epidemiologically to the two previous cases. Despite the lack of evidence of ongoing transmission, a Bong county health official was stationed at the closest point to the community accessible by vehicle (Saint Paul River crossing) to provide mobile telephone updates to the county health team and to arrange safe transport to the ETU for any patients walking out of the community.

On November 3, seven ill Geleyansiesu residents departed the village on foot and were later admitted to the Bong ETU; all tested positive for Ebola virus on November 4, and five died. Each of these seven patients was an immediate family member (two wives and five children) of farmer A or farmer B. Preparations were immediately begun by MOHSW and partners for a full investigation and public health response. Before the investigation could be launched, six additional Geleyansiesu residents were admitted to the Bong ETU; all tested positive for Ebola virus, and five died. Four of these six patients had visited or cared for farmer A after his reported injury or had helped prepare his body for burial; they had not received training on safe burial practices. One of the six was linked to farmer B, whereas the epidemiologic link of one could not clearly be determined.

Investigators returned to the village during November 9–11 to complete case investigations, find and monitor contacts, and conduct active house-to-house case finding. Using MOHSW case definitions, described previously (3), three probable cases and three suspected cases in the village were identified along with 20 contacts. Investigators provided technical assistance to families and local community health volunteers to isolate and treat patients with oral rehydration solutions and facilitate safe evacuation to the Bong ETU for those willing and able to walk out to the ambulance. One of the patients with a probable case of Ebola left to seek diagnosis and treatment at the Bong ETU and tested negative; another died in the village on November 11. One patient with a suspected case went to the Bomi County community care center (4), tested positive, and died. An international partner collected samples on November 11 for the remaining persons with suspected and probable cases, including one post-mortem, and confirmed three cases, all among contacts of farmer A. A clinical partner established isolation tents in the village and was prepared to provide no-touch care to the two remaining cases, but both declined treatment and isolation outside their homes.

What is already known on this topic?

Persons with Ebola virus disease (Ebola) can travel with the infection and spark outbreaks in remote areas. These outbreaks can cause large numbers of illnesses and deaths in the absence of public health interventions to find, isolate, and treat persons with Ebola.

What is added by this report?

In October 2014, CDC, the Liberian Ministry of Health and Social Welfare, and other partners investigated an outbreak in a remote community of Liberia, accessible only by canoe and foot, to confirm the outbreak and begin public health interventions. Although there were delays, ambulance support was established to help those patients who managed to walk out of the community reach an Ebola treatment unit; this intervention removed many patients from the community and contributed to the resolution of the outbreak.

What are the implications for public health practice?

Lessons learned from this outbreak were employed in the planning and interventions for subsequent outbreaks in isolated Liberian communities, improving response times and helping to shorten the course of the outbreak.

On November 19, the clinical partner organization attempted to return to the village to reassess contacts and identify any new cases, but left because of resistance from a group of residents. A meeting with the Gbarpolu County health team and the district’s paramount chief (ranking traditional leader) led to successful reentry into the village on November 29 by MOHSW, CDC, and other partners. During follow-up interviews, it was determined that farmer B had cared for the stepmother of the source patient while she was ill; farmer A had been absent from the village during the weeks before his symptom onset, indicating there were likely two separate Ebola introductions into the village. No new cases were identified during the visit, and both of the previously identified confirmed patients had recovered.

**FIGURE 2:**
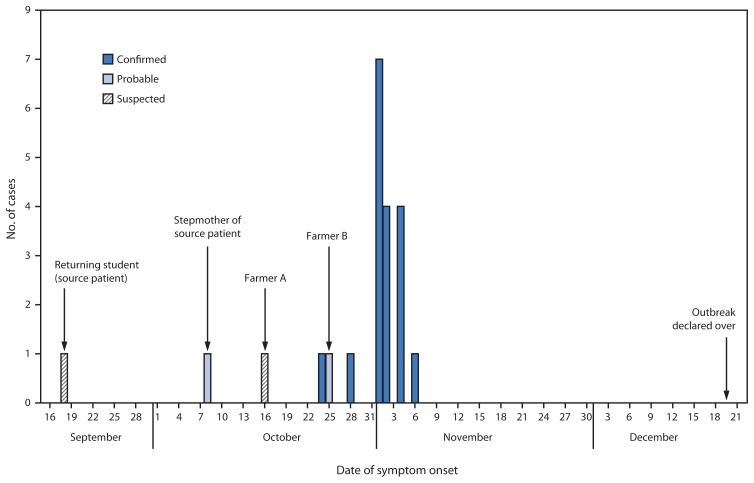
Number of suspected, probable, and confirmed Ebola virus disease cases, by date of symptom onset — village of Geleyansiesu in Gbarpolu County, Liberia, September 18–December 20, 2014

During September 18–November 6, a total of 22 Ebola cases (18 confirmed, two probable, and two suspected) were identified in Geleyansiesu, for an estimated attack rate of 28 cases per 1,000 residents (Figure 2). A total of 16 of the cases were fatal. Median age of patients was 34 years, and six patients (27%) were aged <18 years; 13 (59%) were male (Table). Fifteen of 22 patients were hospitalized at an ETU or community care center, and 18 had a diagnostic test for Ebola completed; samples for 15 were collected at an ETU or community care center, and three were collected in the village. Among those who were hospitalized, the median interval between reported symptom onset and admission to an ETU was 2 days (mean = 3.4 days; range = 2–8 days). Although the patients who became symptomatic before the initial investigation on October 30 generated an average of three secondary cases, no secondary cases were produced by any of the patients who became symptomatic after October 30. On December 20, 21 days after full recovery of the last patient, the outbreak was declared to be over.

### Discussion

During late 2014, multiple outbreaks in remote areas of Liberia were sparked as a result of travelers from affected areas (such as Monrovia) returning to their rural homes. Geleyansiesu is accessible only by a combination of foot and canoe travel, and during this outbreak response, challenges were encountered that have been identified in other rural Liberian counties (5), including poor transportation and communication infrastructure. These challenges, in addition to instances of community resistance to outside intervention, likely delayed and complicated the public health response. A multidisciplinary team including domestic and international partners supported the community in responding to the outbreak, which was effectively controlled with interventions including education about Ebola and establishment of a communication plan to alert health authorities to potential cases and to arrange safe ambulance transportation to an ETU. Rapid response teams can initiate interventions to quickly interrupt Ebola virus transmission, even in remote areas. Flexible support networks, including onsite options for nonambulatory persons and transportation support to link patients to treatment centers, could help limit transmission in remote communities.

## Figures and Tables

**TABLE:** Number and percentage of patients with Ebola virus disease (N = 22), by selected characteristics — village of Geleyansiesu in Gbarpolu County, Liberia, September–November 2014

Characteristic	No.	(%)
**Male**	13	(59)
**Aged <18 yrs**	6	(27)
**Case status**		
Confirmed	18	(82)
Probable	2	(9)
Suspected	2	(9)
**Hospitalized**	15	(68)
**Outcome**		
Dead	16	(73)
Recovered	6	(27)
